# Hearing Aid Use in Older Adults With Postlingual Sensorineural Hearing Loss: Protocol for a Prospective Cohort Study

**DOI:** 10.2196/resprot.9916

**Published:** 2018-10-26

**Authors:** Matthew E Hughes, Joanna Nkyekyer, Hamish Innes-Brown, Susan L Rossell, David Sly, Sunil Bhar, Andrew Pipingas, Alison Hennessy, Denny Meyer

**Affiliations:** 1 Centre for Mental Health School of Health Sciences Swinburne University of Technology Hawthorn Australia; 2 School of Health Sciences Swinburne University of Technology Hawthorn Australia; 3 Bionics Institute Melbourne Australia; 4 Department of Psychiatry St Vincent's Hospital Melbourne Australia; 5 Monash-Alfred Psychiatry Centre The Alfred Hospital Melbourne Australia; 6 Department of Health and Medical Sciences School of Health Sciences Swinburne University of Technology Hawthorn Australia; 7 Department of Otolaryngology University of Melbourne Melbourne Australia; 8 Centre for Human Psychopharmacology School of Health Sciences Swinburne University of Technology Hawthorn Australia

**Keywords:** sensorineural hearing loss, hearing aids, cognition, psychosocial function, speech processing, fMRI

## Abstract

**Background:**

Older adults with postlingual sensorineural hearing loss (SNHL) exhibit a poor prognosis that not only includes impaired auditory function but also rapid cognitive decline, especially speech-related cognition, in addition to psychosocial dysfunction and an increased risk of dementia. Consistent with this prognosis, individuals with SNHL exhibit global atrophic brain alteration as well as altered neural function and regional brain organization within the cortical substrates that underlie auditory and speech processing. Recent evidence suggests that the use of hearing aids might ameliorate this prognosis.

**Objective:**

The objective was to study the effects of a hearing aid use intervention on neurocognitive and psychosocial functioning in individuals with SNHL aged ≥55 years.

**Methods:**

All aspects of this study will be conducted at Swinburne University of Technology (Hawthorn, Victoria, Australia). We will recruit 2 groups (n=30 per group) of individuals with mild to moderate SNHL from both the community and audiology health clinics (Alison Hennessy Audiology, Chelsea Hearing Pty Ltd). These groups will include individuals who have worn a hearing aid for, at least, 12 months or never worn a hearing aid. All participants would be asked to complete, at 2 time points (t) including baseline (t=0) and follow-up (t=6 months), tests of hearing and psychosocial and cognitive function and attend a magnetic resonance imaging (MRI) session. The MRI session will include both structural and functional MRI (sMRI and fMRI) scans, the latter involving the performance of a novel speech processing task.

**Results:**

This research is funded by the Barbara Dicker Brain Sciences Foundation Grants, the Australian Research Council, Alison Hennessy Audiology, and Chelsea Hearing Pty Ltd under the Industry Transformation Training Centre Scheme (ARC Project #IC140100023). We obtained the ethics approval on November 18, 2017 (Swinburne University Human Research Ethics Committee protocol number SHR Project 2017/266). The recruitment began in December 2017 and will be completed by December 2020.

**Conclusions:**

This is the first study to assess the effect hearing aid use has on neural, cognitive, and psychosocial factors in individuals with SNHL who have never used hearing aids. Furthermore, this study is expected to clarify the relationships among altered brain structure and function, psychosocial factors, and cognition in response to the hearing aid use.

**Trial Registration:**

Australian New Zealand Clinical Trials Registry: ACTRN12617001616369; https://anzctr.org.au/Trial/Registration/TrialReview.aspx?ACTRN=12617001616369 (Accessed by WebCite at http://www.webcitation.org/70yatZ9ze)

**International Registered Report Identifier (IRRID):**

RR1-10.2196/9916

## Introduction

### Background

Aging is associated with the onset of postlingual sensorineural hearing loss (SNHL), which refers to hearing loss (or deafness) arising from the pathology of either the inner ear organs or the vestibulocochlear nerve after language acquisition. SNHL accounts for approximately 90% of hearing loss cases in adults and is insidious, progressing from normal (pure tone average [PTA]=0-25 dB) to mild (PTA=26-40 dB), to moderate (PTA=41-70dB), to severe (PTA=71-90 dB), and ending with profound (PTA >91 dB) or total hearing loss. However, more alarming are the sequelae of SNHL that might include rapid cognitive decline [1], impaired psychosocial functioning [2], increased risk of falling [3], and increased risk of incident dementia [4,5]. Recent work, including a meta-analysis of 33 studies [6], reported that SNHL is independently related to both cognitive impairment [7] and the risk of incident dementia [4] and perhaps, most critically, the degree of hearing loss predicts both the degree of cognitive impairment [1] and risk of dementia [4]. Furthermore, recent work suggests that 9% of dementia risk over the life course could be eliminated by avoiding the effects of hearing loss [5].

The scale of these problems is brought into sharp focus in the light of SNHL being the most prevalent chronic condition affecting older adults in developed countries (16%-20%) [8-10] and the second leading cause of years lost to disability globally [11]. Sadly, SNHL often goes undiagnosed and untreated or undertreated [12,13], and the sequelae of SNHL, incident dementia in particular, impose a significant burden not only on individuals and their families and friends but also on national health budgets [14,15]. A recent nationwide study in Australia found that all forms of hearing loss affected 14.5% of Australians (3.6 million people), especially those aged >50 years, with direct costs to the Australian economy of almost Aus $15.9 billion [16]. Furthermore, the number of Australians affected is expected to grow to 18.9% of the population by 2060, thereby slowing or stopping the progression of SNHL is a public health imperative.

The mechanisms underlying the links among cognitive decline, dementia risk, and SNHL are unknown but have been proposed to be due to either common neurodegenerative causes [17,18] or downstream neural changes precipitated by the diminished auditory input [1,19]. Core to these hypotheses is the theory that the diminished auditory input profoundly affects speech processing capabilities and consequently, impairs social functioning [12]. Consistent with these sequelae, individuals with SNHL exhibit accelerated brain atrophy compared with normal hearing adults, especially within right temporal lobe structures [20] that are critical for many cognitive functions. These findings are corroborated by reports that patients with SNHL exhibit atrophic and plastic alteration within cortical brain areas that underpin normal speech processing [21,22].

The initial stages of speech processing in normal hearing adults involve the analysis of basic speech sounds, including phoneme (speech sounds made by the mouth, eg, spoon has 4 phonemes; s/p/oo/n) and acoustic processing [23], which occur within the mid and posterior parts of bilateral dorsal temporal lobe structures, including the superior temporal gyrus, superior temporal sulcus, and planum temporale (for excellent review, see [24]). In their model of speech processing, Hickok et al suggested that subsequent higher-level processing, which includes motor (reproduction and planning) and memory (semantic and linguistic) processes and mapping of the initial sensory and phonological output onto the distinct dorsal and ventral neural pathways [24]. In addition, a left-lateralized dorsal articulatory motor network that includes the inferior frontal, premotor, anterior insula, and temporoparietal cortices maps the output onto articulatory representations; moreover, a bilateral ventral pathway that includes the anterior and posterior portions of the middle and inferior temporal lobes (sulci and gyri) map the output onto ventral lexical and semantic representations to facilitate understanding. Of key interest here are the alterations of brain structures that underlie speech processing in patients with SNHL.

Typically, individuals with SNHL adapt to their altered aural predisposition by relying increasingly on visual cues, including lip-reading. In normal hearing older adults, lip and word reading broadly activate the dorsal articulatory and spectrotemporal and phonological analysis networks mentioned above, except the primary auditory cortices due to the diminished auditory function [25]. Furthermore, SNHL adults engage in a similar network but exhibit higher amplitudes in the attendant structures, especially the prefrontal and premotor cortices, and recruit additional structures, including the right posterior temporal lobe [21,26-28], which is normally only activated by actual sounds [25].

**Figure 1:**
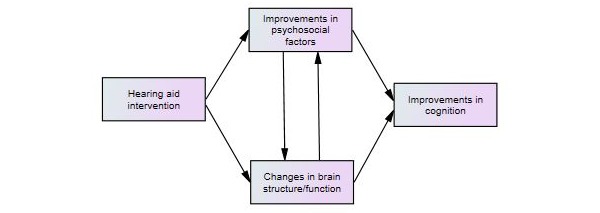
Hypothesized relationships associated with first-time hearing aid usage.

This functional alteration is thought to stem from the loosening of associations between memory and phonological processes and viseme (visual aspects of phoneme pronunciation) processes as SNHL progresses [29] (ie, altered functional connectivity), leading to these plastic brain changes. The phonological processing specialization of the left posterior temporal lobe appears to be preserved, whereas right lateral homologues that are predominantly involved in processing environmental sounds are repurposed to accommodate enhanced visual processing that aids the reading of visual cues (eg, lip-reading) and phonological processing capabilities [21,28,30]. Furthermore, the right anterior part of the superior temporal gyrus that normally performs voice and speaker identification functions is also repurposed for reading visual cues; however, this plastic alteration can be reversed by auditory rehabilitation [31].

Fortunately, a recent investigation suggests that hearing aids worn by individuals with SNHL can preserve or improve auditory and cognitive functioning [32-34] in addition to improving psychosocial well-being [33,35-38]. This suggests that stimulation, as afforded by hearing aid use, can preserve auditory functions and aural specificity and that hearing aids have a protective effect against the deleterious plastic alteration of auditory areas [39].

Alarmingly, however, approximately 90% of people with mild hearing loss, approximately 60% with moderate to severe hearing loss, and approximately 70% aged 65-84 years do not use hearing aids [40]. Furthermore, it often takes about 10 years for an individual to recognize that they have a hearing problem and then act, including obtaining hearing aids, to address this problem [41]. Critically, almost two-thirds of older adults with a hearing impairment do not use hearing aids [42].

This prospective cohort study, in which magnetic resonance imaging (MRI) is utilized in combination with clinical, neuropsychological, and hearing tests, aims to investigate the neurocognitive and psychosocial effects of wearing hearing aids in older adults with SNHL (ACTRN12617001616369).

### Study Objective

Through this study, we seek to understand whether the use of hearing aids can alter the neurocognitive function and affect beneficial plastic brain changes in individuals with SNHL. In particular, we aim to determine whether early intervention can normalize the function of the speech processing brain network. To this end, we will use neuropsychological, clinical, and psychophysical tests in combination with functional and structural MRI (fMRI and sMRI). In addition, fMRI acquisitions will include scanning during the performance of a speech processing task (detailed later) to probe the function of the speech processing network, besides the resting state fMRI to probe the brain network function more generally, whereas sMRI would enable the assessment of both the volume and integrity of the gray and white matter.

### Study Hypotheses

In comparison to the long-term hearing aid users, after wearing a hearing aid for 6 months, the group who have never worn a hearing aid will exhibit improved cognition, reduced depression, improved social interaction, altered activation of the auditory cortices and attendant networks, and altered connectivity between auditory and attendant networks.

In addition, the study will explore any baseline relationships among the cognition, psychosocial, and neural function in participants with and without a hearing aid and any relationships among the improved cognition, psychosocial function, and neural function in participants without a hearing aid after the hearing aid intervention, as seen in Figure 1.

## Methods

### Study Design

The study is a prospective cohort design evaluating the effect of the hearing aid use on cognition, psychosocial factors (eg, depression and social interaction), and neural function. We will recruit 2 participant groups of similar size consisting of people with mild to moderate SNHL. Of note, group randomization is not possible in this study; however, the groups will be matched as much as possible in terms of the degree of hearing loss.

1. Group A: Patients with SNHL who have used hearing aids for, at least, the previous 12 months and plan to continue using their hearing aids for the next 6 months.
2. Group B: Patients with SNHL who have never used hearing aids before and who will be willing to wear hearing aids for the next 6 months.

All participants would be asked to complete, at 2 time points (t) including baseline (t=0) and follow-up (t=6 months), tests of hearing and psychosocial and cognitive function and attend an MRI session.

### Eligibility Criteria

Participants must be aged 55-90 years, speak English as first language, exhibit mild or moderate SNHL with a PTA of thresholds at 0.5-4 kHz in both ears, willing to undergo two 1-hour MRI scanning sessions over a period of 6 months, and willing to wear hearing aids for 6 months or must have been wearing hearing aids for at least 1 year. Furthermore, participants must give written informed consent.

**Figure 2:**
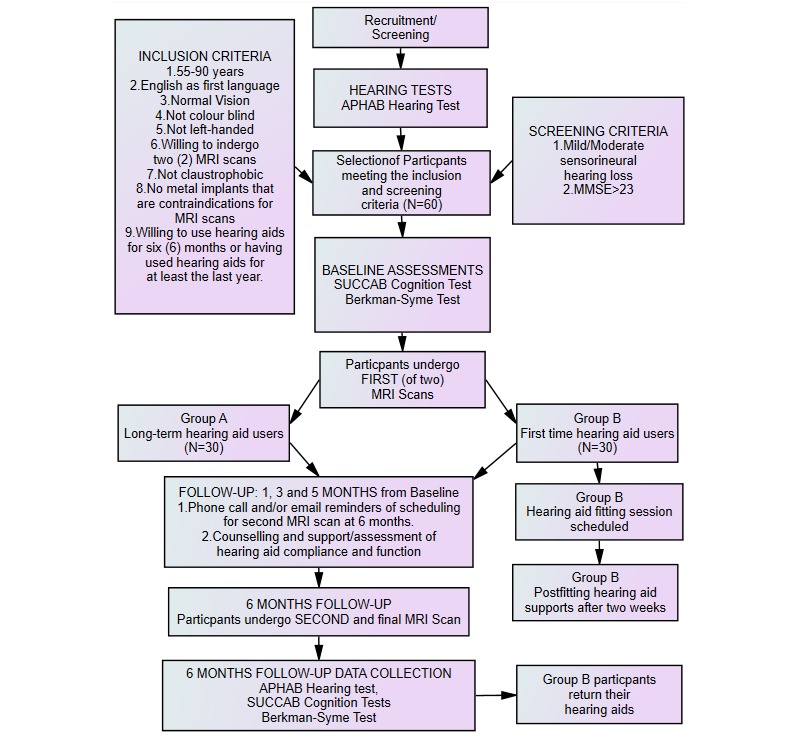
Data collection plan for experimental trial. APHAB: Abbreviated Profile of Hearing Aid Benefit; MMSE: Mini-Mental State Examination; MRI: magnetic resonance imaging; SUCCAB: Swinburne University Computerized Assessment Battery.

Participants will be excluded if they exhibit left handedness, marked visual impairment that would prevent reading, cognitive impairment (defined as a score ≤23 on the Mini-Mental State Examination, MMSE), severe or profound hearing loss, and MRI contraindications. Figure 2 presents the flowchart of the overall data collection plan.

### Recruitment and Screening

Audiology health clinics with existing relationships with the Swinburne University, such as Alison Hennessy Audiology and Chelsea Hearing Pty Ltd, will be contacted to distribute the study promotional material to their clients who have previously undergone hearing assessments at the audiology clinic, been diagnosed with mild or moderate SNHL, and have been wearing their hearing aids for at least 1 year. In addition, the clinic will distribute promotional materials to clients who have recently been recommended to acquire hearing aids but have not yet decided to purchase the aids. All clients who express interest in the study through the audiology clinic will be invited to attend an information session at the Swinburne University of Technology.

Participants who live within the Swinburne community will also be contacted by telephone and email by the researchers to explain the study; participants who express interest will be invited to attend the information session at Swinburne University. At the information session, the researchers will explain the purpose and significance of the study. At the same time, participants will complete a screening questionnaire to identify participants who will be willing to undergo hearing assessments, undergo two 1-hour MRI scans, and fulfill the inclusion criteria. Before completing the screening questionnaire, participants must provide verbal consent. Then, selected participants will be provided with a participant information and consent package for review, which will include further detailed information on the study procedure and a consent form. Participants willing to participate in the study will be scheduled for hearing assessment at the audiology clinics. At the hearing appointments, participants will submit their written consent before their hearing assessments, and those who meet the inclusion criteria will be scheduled to undergo their first MRI scan at a mutually convenient time at the Swinburne University of Technology.

### Study Power Analysis

With 30 consented participants in each group with a significance level of 5%, the power of 80%, and controlling for age, gender, and level of education, the baseline analysis of covariance (ANCOVA) model for spatial working memory performance can detect effect sizes of *d*=0.63 for between-group differences. Allowing 10% attrition in the 6-month follow-up period, a sample size of 27 in each group with a significance level of 5% and power of 80%, a significant group-by-time interaction effect can be detected for a large effect size of *d*=0.77. This study is based on the assumption that an effect size of *d*

### Intervention

#### Fitting of Hearing Aids in Group B Participants

Group B participants will be fitted with two demonstration hearing aids, known as Unitron Tempus Moxi Fit Receiver-in-the-ear hearing aids (Unitron, Kallang, Singapore). The hearing aids will be fitted by professional audiologists at the participating audiology clinics according to the best practice fitting guidelines including real ear (probe tube) measures to verify the amplification and match the appropriate prescribed amplification (typically using the NAL-NL2 prescription developed by the National Acoustics Laboratory [43]) with further adjustment and fine-tuning based on the users’ subjective preferences. In addition, the study audiologists will provide a pamphlet guide on the hearing aid use as take-home materials as well as a verbal explanation on how to use these hearing aids. Furthermore, postfitting support will be provided after 2 weeks to make sure that each participant is progressing with their hearing aids.

#### Follow-Up Periods After the Baseline Data Collection

During the 6-month waiting period between MRI scans, phone call and email reminders for the second testing session will be sent to all participants at 1, 3, and 5 months. At the same time, counseling and other compliance-improving policies [44-46] will be provided to ensure that participants are wearing their hearing aids. In addition, follow-up checks will be conducted by the audiology clinics every 6 weeks for first-time hearing aid users, during which hearing aid usage data will be downloaded and hearing aids will be restarted. An automatic internet-based data logging function installed in the hearing aids will be used to collect hearing aid usage data, which will be used to monitor and assess hours of hearing aid use by these participants. Furthermore, all participants will be encouraged to set their own goals for the hearing aid use and will be asked to assess how well these goals have been met on a regular basis.

#### After the 6-Month Follow-Up Period

All participants will return to the audiology clinics for further hearing assessments. Group B participants who received loaned hearing aids will return them. In addition, hearing aid supplier information will be provided to them if they are ready to purchase hearing aids. After hearing assessments, participants will return to the Swinburne University to complete the 6-month follow-up assessments.

### Contingency Plan

Participants who decide to withdraw because of some discomfort experienced with hearing aids or do not undergo a second MRI testing will be retained in the study for baseline MRI analyses only. In addition, intention-to-treat (ITT) analyses will be used for other analyses not involving MRI data.

### Additional Costs and Reimbursements

No costs would be associated with participating in this research project other than transport costs. All participants will be reimbursed Aus $60 to cover these costs after the completion of the 6-month testing protocol.

### Outcomes

#### Assessments for Study

The assessments selected in this study are categorized as follows: hearing assessments, demographic questionnaire, hearing aid benefit questionnaire, cognitive performance, mood and social interaction assessments, and MRI scanning. All participants enrolled in this study will complete these assessments at the baseline and again after 6 months.

##### Hearing Assessments

**Otoscopy and Tympanometry**

Following otoscopy, all participants will undergo tympanometry and acoustic reflex testing to assess the status of the middle ear [47,48].

###### Pure Tone Audiometry in Each Ear

To understand the degree of hearing impairment, and classify participants according to the type of hearing loss, hearing ability will be measured at threshold frequencies of 0.25, 0.5, 1, 1.5, 2, 3, 6, and 8 kHz (air conduction) and 0.5, 1, 2, and 4 kHz (bone conduction) in both ears. The choice of frequency to be tested corresponds to the amplification range of most modern hearing aids and is consistent with the capturing sensitivity at frequencies affected by SNHL and noise-induced damage. Only participants with either mild or moderate SNHL will be included in this study [47,48].

##### Demographics

Data on a variety of demographic variables will be collected to describe the characteristics of study participants.

##### The Abbreviated Profile of Hearing Aid Benefit Inventory

The Abbreviated Profile of Hearing Aid Benefit (APHAB) inventory [49], which is a 10-minute self-assessment inventory, will be used to assess the hearing aid benefit (for normative data, see [50]). The 4 scales of the APHAB will be assessed, namely ease of communication (EC) in favorable environments, ease of communication with background noise (BN), ease of communication with acoustic reverberation (RV), such as listening to sounds in a large room, and aversiveness (AV), which measures negative reactions to environmental sounds such as traffic and alarm bells

The APHAB inventory subscales exhibit acceptable reliability with Cronbach alpha value of .87 (EC), .83 (RV), .82 (BN), and .86 (AV) in unaided conditions, and test-retest correlation coefficients of .80, .65, .71, and .89, respectively [49].

##### Cognitive Assessments

###### The MMSE Questionnaire

MMSE will be used to assess cognition and will be administered to test the cognitive functioning. MMSE is a valid and reliable way of globally assessing a limited range of cognitive functions [51]. This examination will test the following 5 areas of the cognitive function: orientation, registration, attention and calculation, recall, and language. Participants who exhibit a confusion state while completing the MMSE questionnaire will be advised that they cannot be included in the trial and will be advised to see their general practitioner.

###### The Swinburne University Computerized Assessment Battery

The Swinburne University Computerized Assessment Battery (SUCCAB) will be used to assess the cognitive performance. This cognitive test battery will allow the assessment of changes in the cognitive performance of all participants over a period of 6 months. The battery will test contextual memory, immediate recognition, simple reaction time, choice reaction time, congruent stroop, incongruent stroop, spatial working memory, and delayed recognition memory. The reliability and validity testing of this battery has demonstrated that SUCCAB is sensitive to aging and has been shown to be particularly effective for measuring short-term changes in cognition of the elderly [52]. Furthermore, SUCCAB correlates strongly with memory subsets in the Wechsler Adult Intelligence Scales [53].

##### Psychosocial Assessments

###### Depression, Anxiety, and Stress Scale

Depression, Anxiety, and Stress Scale (DASS) is a well-established self-rating mood scale for measuring 3 related negative emotional states of depression, anxiety, and stress. To assess changes in mood in this study, DASS-21, which is a shortened version (21 items) of the full DASS (42 items), will be used [54]. DASS-21 exhibits excellent subscale reliability with Cronbach alpha of .94 for depression, .87 for anxiety, and .91 for stress [55] and has been validated against other well-established measures, including the Beck Depression Inventory, the Beck Anxiety Inventory, and the State-Trait Anxiety Inventory [55].

###### The Berkman-Syme Social Network Index

The Berkman-Syme Social Network Index (SNI) [56] will be used to assess participants’ social interaction and connections with families and friends. In this study, 12 types of social relationships will be assessed, namely relationships with a spouse, parents, parents-in-law, children, other close family members, close neighbors, friends, workmates, schoolmates, fellow volunteers, members of groups without religious affiliation, and religious groups. Although SNI is commonly used in epidemiological research [57], no detailed assessments exist of its reliability, even if the originators reported an overall value of .92 in a 14-week follow-up study of 245 first-year university students [56]. Because SNI relies on self-report, its validity relies on the honesty of participants.

##### Magnetic Resonance Imaging Assessments

###### Speech Processing Task

During fMRI scanning, participants will view a series of human faces (1 male and 1 female actor) that mouth words in blocks (or “epochs”) lasting 16 seconds (1 word per 2 seconds) each. Each block will be one of 4 conditions termed *matched* (MAT), *mismatched* (MIS), *no sound* (NOS), and *control* (CON), preceded and followed by 10-second rest periods, where a fixation cross (“+”) will be displayed. In each of 2 scanning runs, 4 repetitions of each block type (16 total blocks per run) will be presented in a pseudorandom order, each utilizing different word stimuli. The stimulus presentation time for each of the two scanning runs will be 426 seconds (7 minutes 6 seconds); an additional 20 seconds of imaging data will be acquired following the end of the stimulus presentation to allow the hemodynamic response to return to the baseline.

During MAT, the faces will mouth single syllable, high-frequency words (visual stimuli), such as “cat” or “house,” and the corresponding audio input (auditory stimuli) will be played through the headphones. During MIS, the stimuli will remain the same, but the mouthed words and auditory stimuli will be semantically unrelated (eg, “cat” is mouthed but “house” is heard). During NOS, the visual stimuli will be presented but not the auditory stimuli. During CON, the faces will be presented but will not mouth the words; instead, they will simply open and close the mouth without auditory stimuli. All participants will be asked to press a button whenever a face appears to ensure participant attendance to the task.

###### Magnetic Resonance Imaging Scan Acquisition

The MRI scanning session will include the acquisition of 4 different types of scan data while participants lie supine in the scanner wearing MRI-compatible OptoActive headphones (OptoAcoustics). Participants will have either normal or corrected to normal vision using MRI-compatible goggles. These acquisitions will include 2 fMRI scanning runs while participants perform the speech processing task, a high-resolution T1-weighted structural image (∼8 minutes), diffusion-weighted images (∼10 minutes), and a resting state fMRI scanning acquisition (∼10 minutes); the total scanning time will be 42 minutes.

The following details the different scan protocols, including scan parameters, preprocessing, and data analysis:

1. Scan acquisition for the speech processing task will utilize a T2* sensitive echo-planar imaging sequence (repetition time [TR]=2000 ms; echo time [TE]=30 ms; flip angle=90°; field-of-view=192 mm, 46 interleaved slices, 3-mm^3^ isotropic voxels). Preprocessing and statistical analysis will be performed using SPM12 and associated toolboxes. Preprocessing will include slice-timing correction, motion correction, coregistration of realigned functional images to structural (T1-weighted) scans, warping (“normalization”) of structural and functional scans into standardized stereotactic space, and spatial smoothing of functional images. The data will be modeled by constructing separate regressors that depict the onset and duration of MAT, MIS, NOS, and CON blocks, convolved with the canonical hemodynamic response function supplied with SPM12. Covariates of no interest (eg, image realignment and other noise parameters) will model noise components.
2. Resting state scanning will utilize T2*-weighted images, which will be acquired continuously using an interleaved multiband sequence (multiband acceleration factor=6; bandwidth=1860 Hz/Px; TR=870 ms; TE=30 ms; echo spacing=0.69 ms; flip angle=55°; field-of-view=192 mm; voxel resolution=2 mm x 2 mm x 2 mm; slice-thickness=2 mm; number of slices=66). In addition, multiband acquisition sequences will be derived from the Human Connectome Project [58]. Data analysis will be performed using the “CONN” connectivity toolbox [59] to test changes in the functional connectivity between brain areas we find to be critical in the sensory integration task as a function of wearing the hearing aids, in addition to broader network connectivity. Next, images will be realigned, normalized to a standard stereotactic space defined by the Montreal Neurological Institute (MNI space), spatially smoothed with a 5-mm kernel, and temporally band-pass filtered (0.010-0.100 Hz). T1-weighted images will be segmented into gray and white matter, as well as the cerebrospinal fluid. Then, physiological noise and motion parameters will be regressed from the functional images using ACompCor [60]. Temporal confounds regressed from the time series will include head motion parameters and their temporal derivatives, in addition to ACompCor-derived noise components.
3. The T1-weighted image will be acquired using a magnetization-prepared gradient echo sequence (TR=1900 ms; TE=2.52 ms; flip angle=9°; field-of-view=256 mm x 256 mm; 176 slices; 1-mm^3^ voxels). Diffusion-weighted images will be acquired using an isotropic diffusion tensor imaging sequence for fractional anisotropy (FA) estimations (number of directions=60; *b*-value=3000 s/m^2^; slice-thickness=2.5 mm; TR=8400 ms; TE=117 ms; flip angle=90°). In addition, T1-weighted images will be used in the coregistration of functional data and also perform the analysis of regional brain volumes using voxel-based morphometry (VBM) using DARTEL procedures. For VBM, images will be manually reoriented and segmented [61], then a template will be created from the reoriented images using the nonlinear deformations that best align the segmented images, which will subsequently be warped into stereotactic space and spatially smoothed.
4. Finally, we will perform diffusion-weighted magnetic resonance (MR) white matter tractography using “MRTrix” [62] to assess white matter tract changes as a function of wearing the hearing aids. Preprocessing steps will include constructing a brain mask, estimating diffusion tensor components, and performing constrained spherical deconvolution. Subsequently, we will perform whole-brain and seed-based fiber tracking.

###### Auditory Stimuli Input Considerations

As the speech processing task will involve hearing word stimuli, the auditory input for each participant will be tailored to fit a normalized audiogram, that is, the gain will be enhanced at impaired frequencies. This will be performed by fitting a spline function to prerecorded audiograms that will be used to modulate auditory stimuli for the left and right ears separately. Additionally, the headphone output will be modified such that it is consistent across individuals.

### Primary Statistical Analyses

In this study, repeated measures mixed model group (no hearing aid vs hearing aid users) x Time (Time 1 vs Time 2) analysis (RMMM) will be used for all analyses. The missing data will be accommodated in this analysis; however, in the case of whole-brain fMRI analyses, only completed protocol participants will be included. In the ITT RMMM analyses, the autoregressive (AR) dependence will be assumed.

#### Cognitive Data

The SUCCAB performance measure for spatial working memory will be used as the primary SUCCAB measure; this measure has been found to be particularly effective in measuring short-term changes in cognition of the elderly [52] and is calculated by dividing the response accuracy by the reaction time for a spatial working memory task. Using this and other SUCCAB performance measures, baseline values for the 2 groups will be compared using an ANCOVA analysis, controlling for age, gender, and education level. In addition, changes in these values over time will be compared for the 2 groups of respondents using an ITT RMMM, controlling for age, gender, and education level and any variable that differs markedly between the groups at the baseline.

**Mood and Social Interaction Data**

In this study, the mood will be assessed using the DASS scale, and social interaction will be measured using the Berkman-Syme SNI. The baseline measures for the 2 groups will be compared using analysis of variance. In addition, changes in these measures over time will be compared for the 2 groups of respondents using an ITT RMMM, controlling for age, gender, and education level and any variable that differs markedly between the groups at the baseline.

**Neuroimaging Data**

Inferences from functional and structural neuroimaging analyses will be assessed using the random field theory to correct for multiple comparisons at the cluster level.

##### Speech Processing Task Data: Functional Alteration

First, we will compute the contrast of MAT>NOS (NOS controls for viseme processing) for each participant and enter the contrasts into an RMMM to assess the effect of the first-time hearing aid use on speech sound processing; (2) we will assess changes in the functional connectivity in key areas determined from this analysis using the generalized psychophysiological analytic approach [63]; (3) finally, we will use key areas of difference as seeds in the functional connectivity analysis of the resting state data.

Secondly, we will compute the contrast of NOS>CON (CON controls for basic face motion processing) for each participant and enter the contrasts into an RMMM to assess the effect of the hearing aid use on viseme processing; (2) we will assess changes in the functional connectivity in key areas determined from this analysis using the generalized psychophysiological analytic approach [63]; (3) finally, we will use key areas of difference as seeds in the functional connectivity analysis of the resting state data.

##### Structural T1 Data: Structural Alteration

To assess plastic alteration in response to the first-time hearing aid use, these spatially smooth gray matter images will be entered into an RMMM.

### Exploratory Analyses

#### Cognitive Data

Correlations between the SUCCAB performance measures and neuroimaging data will be investigated at the baseline and after 6 months for each o group using ANCOVA analyses and ITT Hierarchical Linear Model analyses.

#### Psychosocial Data

Correlations between DASS and the Berkman-Syme SNI with the SUCCAB performance measures and the neuroimaging data will be investigated at the baseline and after 6 months for each group using ANCOVA analyses and ITT Hierarchical Linear Model analyses. In addition, structural equation modeling will be used to explore the role of the mood and social interaction data as process variables for the effects of hearing aid use on the cognition and neural function, testing the hypothesized model shown in Figure 1.

#### Neuroimaging Data

We will explore changes in phoneme and viseme processing separately and their integration, as a function of the hearing aid use by modeling combinations of MAT, MIS, NOS, and CON, in addition to any change in the functional connectivity using the generalized psychophysiological analytic approach [63]. In addition, we will explore the altered whole-brain connectivity and internetwork coupling using the resting state data. Finally, we will assess the white matter alteration using the VBM approach described above for the gray matter. Furthermore, we will plan to explore changes in the white matter integrity using diffusion tensor analyses and diffusion tractography.

## Results

The speech processing task was programmed and tested during September 2017-December 2017. Training of research staff on research protocols (cognitive, hearing, and MRI session testing) was conducted intermittently between February 2016 and February 2020. In addition, the baseline testing sessions will commence in February 2018 and will be completed by June 2020, and the follow-up sessions will be completed by December 2020. Furthermore, baseline session data analyses will be completed by October 2019, and final longitudinal data analyses will be completed by July 2020.

## Discussion

### Summary

SNHL is strongly associated with cognitive decline, social and mental health problems, and incident dementia. SNHL leads to brain atrophy and neuroplasticity that may be detrimental to auditory rehabilitation. Some evidence indicates that the use of hearing aids may slow or improve this pathology. In this retrospective cohort study, we utilize cognition and psychosocial testing in combination with structural and functional neuroimaging to assess the impact of the hearing aid use on the neurocognitive function and brain structure in those with SNHL. To the best of our knowledge, this is the first study to directly assess structural and functional brain changes arising from the use of hearing aids in older adults with SNHL. Currently, there is a paucity of neuroimaging studies in the SNHL field generally, which is surprising given what is known about neural plasticity in SNHL. A chief motivation for this work is to address this shortcoming, yielding critical data for SNHL research and ideally, may prompt greater use of hearing aids in those with SNHL.

### Limitations

This study has some limitations that must be addressed. There are numerous aspects of speech processing, in general, and its impairment in SNHL. In this study, we have chosen to examine the processing of one aspect alone, namely monosyllabic word processing. This approach was selected to make the task easy to perform for participants and to ease data interpretation. Hence, our analyses will not reveal all aspects of speech processing dysfunction in SNHL such as sentence comprehension [64]. In addition, studies examining the cognitive impairment in SNHL have not utilized consistent neuropsychological testing protocols; hence, the component processes probed across studies might not be consistent, inhibiting the generalization of findings across studies. However, here we use a standardized battery that has been found to be particularly sensitive in older adults [52].

### Conclusions

SNHL is a major and growing health problem for older adults that touches most aspects of their lives, especially their cognitive function, mental health, and well-being. The use of hearing aids enhances the lives of these individuals through not only enhanced hearing but also improved social interaction, mood, and cognitive functioning. Such day-to-day functional enhancement in individuals with SNHL suggests that beneficial plastic changes occur in their brains as a consequence of hearing aid use; however, the use of hearing aids among this population is low.

## Abbreviations

ANCOVA: analysis of covariance
APHAB: Abbreviated Profile of Hearing Aid Benefit
AV: aversiveness
BN: background noise
CON: control
DASS: Depression, Anxiety and Stress Scale
EC: ease of communication
FA: fractional anisotropy
fMRI: functional magnetic resonance imaging
HA: hearing aid group
ITT: intention-to-treat
MAT: matched
MIS: mismatched
MMSE: Mini-Mental State Examination
MNI: Montreal Neurological Institute
MR: magnetic resonance
MRI: magnetic resonance imaging
NOS: no sound
PTA: pure tone average
RMMM: repeated measures mixed model
RV: reverberation
sMRI: structural magnetic resonance imaging
SNHL: sensorineural hearing loss
SNI: Social Network Index
SUCCAB: Swinburne University Computerized Assessment Battery
TE: echo time
TR: repetition time
VBM: voxel-based morphometry
